# Quantum coherence in a processable vanadyl complex: new tools for the search of molecular spin qubits†
†Electronic supplementary information (ESI) available: Full experimental section and methods. Additional characterization including: AC susceptibility data (Fig. S1–S5); CW- and pulsed EPR results (Fig. S6–S11); STM and XPS of the UHV deposition (Fig. S12–S14); calculated field dependence of the eigenvectors composition (Fig. S15). See DOI: 10.1039/c5sc04295j


**DOI:** 10.1039/c5sc04295j

**Published:** 2015-12-11

**Authors:** Lorenzo Tesi, Eva Lucaccini, Irene Cimatti, Mauro Perfetti, Matteo Mannini, Matteo Atzori, Elena Morra, Mario Chiesa, Andrea Caneschi, Lorenzo Sorace, Roberta Sessoli

**Affiliations:** a Department of Chemistry “Ugo Schiff” , University of Florence & INSTM RU of Florence , Via della Lastruccia 3-13 , 50019 Sesto Fiorentino , Italy; b Department of Chemistry , University of Turin & NIS Centre , via P. Giuria 7 , 10125 , Torino , Italy . Email: roberta.sessoli@unifi.it ; Email: lorenzo.sorace@unifi.it

## Abstract

Electronic spins in different environments are currently investigated as potential qubits, *i.e.* the logic units of quantum computers. These have to retain memory of their quantum state for a sufficiently long time (phase memory time, *T*_m_) allowing quantum operations to be performed. For molecular based spin qubits, strategies to increase phase coherence by removing nuclear spins are rather well developed, but it is now crucial to address the problem of the rapid increase of the spin–lattice relaxation rate, *T*_1_^–1^, with increasing temperature that hampers their use at room-temperature. Herein, thanks to the combination of pulsed EPR spectroscopy and AC susceptometry we evidence that an evaporable vanadyl complex of formula VO(dpm)_2_, where dpm^–^ is the anion of dipivaloylmethane, presents a combination of very promising features for potential application as molecular spin-qubit. The spin–lattice relaxation time, *T*_1_, studied in detail through AC susceptometry, decreases slowly with increasing temperature and, more surprisingly, it is not accelerated by the application of an external field up to several Teslas. State-of-the art phase memory times for molecular spin systems in protiated environment are detected by pulsed EPR also in moderate dilution, with values of 2.7 μs at 5 K and 2.1 μs at 80 K. Low temperature scanning tunnel microscopy and X-ray photoelectron spectroscopy *in situ* investigations reveal that intact molecules sublimated in ultra-high vacuum spontaneously form an ordered monolayer on Au(111), opening the perspective of electric access to the quantum memory of ensembles of spin qubits that can be scaled down to the single molecule.

## Abstract 2

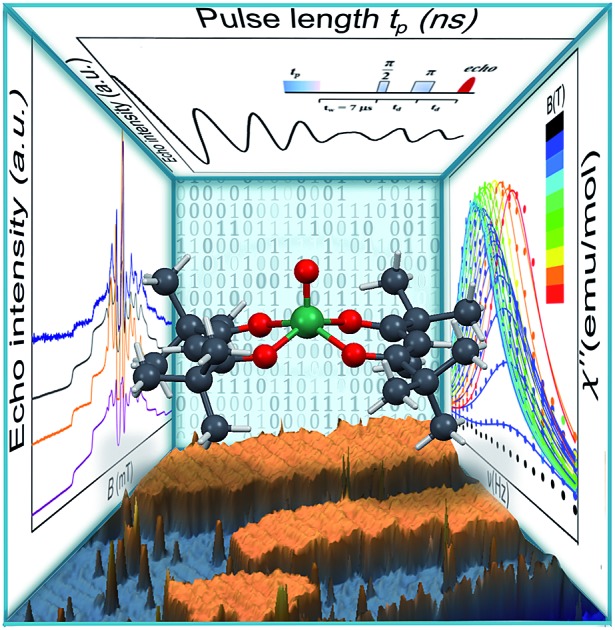
A multitechnique investigation of an evaporable vanadyl spin system with long-lived quantum coherence that self-assembles on gold.

## Introduction

The realization of a quantum computer is expected to trigger a second revolution in information and communication technology,^1^–^4^ allowing for unequalled computation capabilities in disparate fields, ranging from structural biology^5^ to quantum physics.^3^ Quantum bits, or qubits, are at the basis of quantum computation, and different strategies to realize them are currently explored,^6^ including ionic traps,^7^ quantum dots in semiconductors,^8^,^9^ photons,^10^ and superconducting nanostructures.^11^ Spins, either nuclear^12^–^14^ or electronic,^15^–^19^ are among the most efficiently addressable targets to build these logical units, as their initialization and read-out can be performed by well-established magnetic resonance techniques. The parameters to be optimized in the design of these qubits are: i) the longitudinal relaxation time, *T*_1_, which corresponds to the lifetime of a classical bit that can assume either the |0> or the |1> value; ii) the characteristic time in which the spin loses the memory of the phase of the superposition state in which it has been prepared. A lower estimation of this decoherence time, *T*_2_, can be extracted by the memory time, *T*_m_, which is commonly measured with pulsed EPR or NMR: the ratio of *T*_m_ over the time necessary for an individual quantum operation has to be larger than 10^4^ to allow for fault tolerant quantum computing.

In the field of electron spin-based qubits nitrogen vacancies in diamond^20^,^21^ and impurities in silicon and silicon carbide^22^ exhibit long-lived quantum coherence but present major challenges in the control of their organization and coupling to perform quantum logic operations. Molecular spin based qubits, on the contrary, can be organized on surfaces and the interaction between them tuned at will through a rational synthetic design. After an extensive research on polynuclear transition metal complexes^16^,^23^,^24^ optimized to exhibit a long *T*_m_, the research in this field has recently focused back on the simplest spin *S* = 1/2 systems constituted either by organic radicals^25^ or by 3d transition metal ions.^26^–^28^ These have relatively long *T*_m_, in particular at high temperature, because there are no excited spin levels that can foster the magnetic relaxation when thermally populated. In these systems the interaction of the electronic spin with the nuclear spins is the most relevant source of decoherence. Outstanding results have very recently been obtained with vanadium(iv) ions assembled with nuclear spin-free ligands.^27^ When magnetic dilution is made in a nuclear spin-free solvent, such as CS_2_, *T*_m_ approaches one millisecond at low temperature,^29^ showing that molecular spin systems can be as performant as extended inorganic structures. Unfortunately this remarkable long coherence is rapidly lost on increasing temperature because the spin–lattice relaxation acts as a limiting factor for *T*_2_.^30^ A recent investigation has clearly evidenced that solid crystalline solutions can enhance *T*_1_, thus resulting in enhanced coherence time at high temperature^26^ but the mechanisms of relaxation, as well as strategies to enhance *T*_1_, are still poorly investigated.

In this study we have investigated the magnetic relaxation of a simple mononuclear complex of vanadium(iv) by the combination of AC magnetic susceptometry to study spin–lattice relaxation with pulsed EPR spectroscopy to characterize the spin coherence. The two techniques can in fact shed light on different contributions to the relaxation but their association is unprecedented in the search for potential spin-based qubits.

The vanadyl complex VO(dpm)_2_, where dpm^–^ is the anion of dipivaloylmethane, has been selected because the strong V=O bond is expected to increase the rigidity of the coordination sphere with a reduction of spin–lattice relaxation efficiency. The presence of β-diketonate ligands in a neutral complex imparts a high volatility that can be exploited to deposit the molecule on surfaces. An unexpected long *T*_1_ over wide field and temperature ranges has been found to accompany *T*_m_ values that are among the longest ones observed in molecular species surrounded by spin active nuclei. The *in situ* morphological and spectroscopic characterization of a monolayer deposit of VO_2_(dpm)_2_ on Au(111) suggest that the molecules are intact on the surface, making these simple units potential candidates as molecular qubit individually addressable by scanning probe techniques.

## Results and discussion

**Fig. 1:**
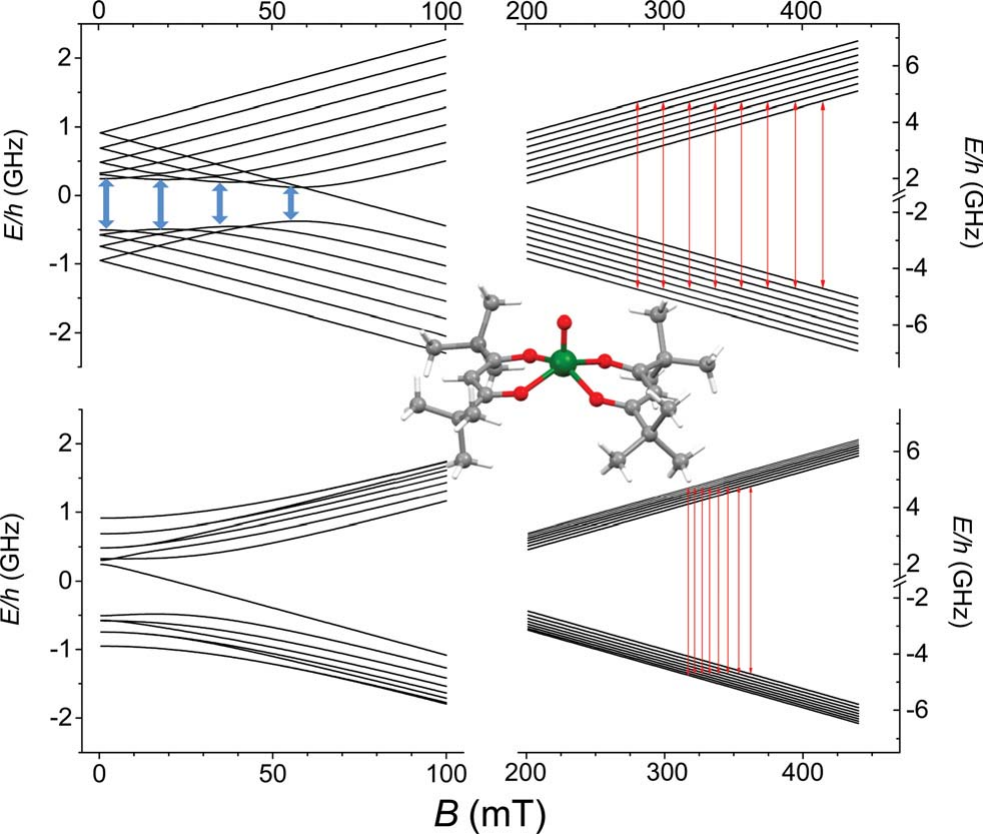
Zeeman splitting of the *S* = 1/2, *I* = 7/2 manifold calculated with the field applied along the largest hyperfine coupling component (upper) and along the smallest (lower) (parameters in the text). Red lines correspond to the observed X-band (*ν* = 9.62 GHz) EPR transitions while in pale blue are drawn the potential low frequency transitions at the avoided level crossings. In the inset the molecular structure of VO(dpm)_2_.

The synthesis of crystalline VO(dpm)_2_ was achieved according to an earlier reported procedure,^31^ operating under inert atmosphere to avoid oxidation. VO(dpm)_2_, prepared in the crystalline form and characterized by X-ray diffractometry (see ESI†), presents a typical square pyramidal coordination (Fig. 1) with a short V=O double bond (1.59 Å *vs.* an average of 1.964 Å for the V–O single bonds). Deviations from tetragonal symmetry are already visible in the first coordination sphere in both bond lengths and bond angles. Given that the system crystallizes in the monoclinic *P*2_1_ space group, two sets of molecules with the V=O directions forming an angle of 64.1° are present in the crystal lattice. The strongly axial ligand field produced by the short V=O bond removes orbital degeneracy with the d_*xy*_ orbital being the lowest in energy and the only one to be half occupied. Vanadyl systems are therefore well described by a spin *S* = 1/2 with slightly anisotropic ***g*** tensor close to the free electron value. The most abundant isotope of vanadium, ^51^V (99.75%), is characterized by *I* = 7/2 thus the *S* = 1/2 doublet is further split in 16 states by hyperfine interaction as schematized in Fig. 1.

### Magnetization dynamics

The magnetization dynamics, investigated by AC-susceptometry (see ESI†), of a polycrystalline sample of VO(dpm)_2_, hereafter **1**bulk, revealed no imaginary component of the susceptibility in zero static field down to the lowest investigated temperature (1.9 K). The application of a weak field induced however slow relaxation of the magnetization with the concomitant decrease of the real component *χ*′ and the appearance of a peak in *χ*′′ component (ESI Fig. S1†). In a field of 0.2 T the entire magnetization of the system relaxed slowly and this field was selected to investigate the temperature dependence of the relaxation time.

**Fig. 2:**
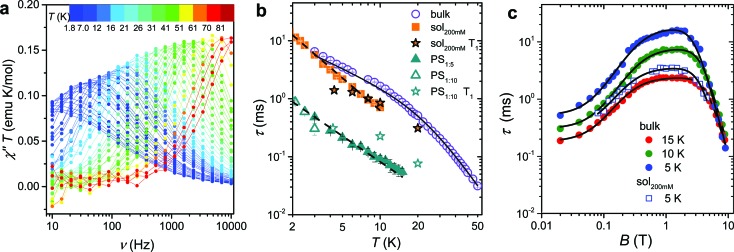
(a) Frequency dependence of the imaginary component of the AC susceptibility of **1**bulk in *B*_dc_ = 0.2 T multiplied by temperature to be readable in the whole 2–80 K temperature range. (b) Temperature dependence of the relaxation time of the magnetic susceptibility measured in *B*_dc_ = 0.2 T for the pure and the diluted samples of **1**: two dispersions in polystyrene with 1 : 5 and 1 : 10 mass ratio and a 200 mM CH_2_Cl_2_ : toluene frozen solution (see legend). The black solid line corresponds to the best-fit of **1**bulk data using eqn (1), while the broken lines correspond to simulation with *T*^–*n*^ law. *T*_1_ for the diluted sample, extracted from pulsed EPR spectra, are shown for comparison. (c) Field dependence of the relaxation time of the magnetic susceptibility of **1**bulk and **1**sol_200 mM_ frozen solution sample (see legend). The solid lines represent the best fit obtained with eqn (3). Best fit parameters are reported in Table 1.

Maxima in *χ*′′ were observed up to 80 K for frequencies lower than 10 kHz, as shown in Fig. 2a (ESI Fig. S2† for *χ*′), evidencing also a gradual increase in the width of the distribution on lowering the temperature (see ESI and Fig. S3†). Such a high temperature slow relaxation is usually observed in molecules exhibiting strong magnetic anisotropy known as Single-Molecule Magnet (SMMs), for instance in double-decker TbPc_2_ complexes,^32^ but clearly it has a different origin here. The data, reproduced with the Debye model,^33^ allowed to extract the relaxation time of the susceptibility, *τ*, reported in Fig. 2b. It is evident that *τ* does not follow the Arrhenius behaviour, in agreement with the lack of electronic/magnetic states that can be thermally populated providing a path for the multiphonon Orbach mechanism of relaxation.^34^,^35^ On the contrary the temperature dependence of *τ* can be reproduced by considering different contributions to the relaxation rate:

1 *τ*^–1^ = *aT* + *bT*^*n*^

where the first term (*a* = 59 ± 2 s^–1^ K^–1^) corresponds to the direct mechanism, dominating at low temperature, and the second one (*b* = 0.052 s^–1^ K^–*n*^) to a Raman-like, *i.e.* a multiphonon process involving virtual excited states.^34^ Interestingly the exponent *n* = 3.22 ± 0.02 is much smaller than the value of 9 or higher expected for the Raman process,^35^ but approaches the value of 3 predicted in the case that both acoustic (lattice) and optical (molecular) vibrations are involved in the process.^34^

To shed light on the mechanisms of magnetic relaxation the AC susceptibility was investigated in a wide field range, *i.e.* up to 8.8 T, for three different temperatures, 5, 10, and 15 K. Notice that in this temperature range the direct process dominates as indicated by the almost linear dependence of *τ*^–1^ on *T*. The corresponding relaxation times are reported in Fig. 2c. The initial increase of *τ* for weak applied field is followed by an almost flat region that extends up to *ca.* 4 T, followed by a rapid decrease at higher fields. Data of Fig. 2c were reproduced considering different relaxation mechanisms that can be active in *S* = 1/2 systems. According to the seminal work done by de Vroomen *et al.* on the Cu^2+^ Tutton salt,^36^ two contributions to the relaxation rate can be considered:

2 *τ*^–1^ = *τ*_Z_^–1^ + *τ*_int_^–1^

The first term represents the direct mechanism between the two states split by the Zeeman energy, which is expected to vanish in zero field as a result of the Kramers theorem.^37^ In fact, a pure and isolated *S* = 1/2 should not be able to relax in zero field. The second term takes into account a sort of internal field whose origin can be either intramolecular (*i.e.* hyperfine interactions) or intermolecular (*i.e.* due to dipolar or exchange interactions). The latter is responsible for the efficient relaxation in zero field and presents, for the direct mechanism,^38^ a field dependence that is similar to the Brons-van Vleck formula developed to describe the Raman process in concentrated systems.^39^ Summing up the two contributions to the field dependence in eqn (2) the data of Fig. 2c have been reproduced according to:
3

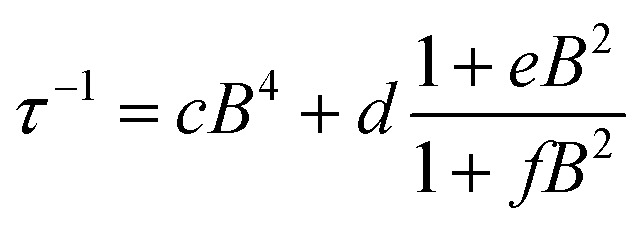




The first term is the typical field dependence of the direct process for a *S* = 1/2 spin and reflects the fact that the larger is the Zeeman splitting between the states the higher is the density of phonons matching it. In the second one the *d* term represent the zero field relaxation rate, similar to the tunnelling rate in SMMs,^33^ the *f* parameter takes into account the ability of the external field to suppress these mechanisms, while the *e* parameter, strongly dependent on the concentration of the spin centres, takes into account the field effects on the relaxation of interacting spins.^39^ The best-fit values obtained with eqn (3) are summarized in Table 1. The *c* parameter for *T* = 5 K should be considered with caution because only a small fraction of the susceptibility is detected at such high fields. The field range where the relaxation remains slow is really remarkable, suggesting that the direct mechanism of relaxation is not very efficient.

**Table 1:** Best-fit parameters of eqn (3) used to reproduce the field dependence of the magnetization relaxation rate of **1**bulk measured at the three investigated temperatures and of **1**sol_200 mM_ at 5 K (last row)

*T* (K)	*c* (T^–4^ s^–1^)	*d* (s^–1^)	*e* (T^–2^)	*f* (T^–2^)
5	0.96 ± 0.04	2060 ± 180	7.6 ± 0.7	240 ± 30
10	0.73 ± 0.01	3460 ± 150	7.6 ± 0.4	179 ± 12
15	0.97 ± 0.03	5740 ± 270	15.1 ± 0.9	190 ± 16
5	2.00 ± 0.11	3600 ± 550	13.7 ± 1.4	160 ± 40

In ESI Fig. S3 and S4† we also report the temperature and field dependence of the width of the distribution of relaxation rate, respectively. The width is correlated to the parameter *α* of the extended Debye formula (see ESI†). In general the distribution is quite narrow increasing up to 0.3 at the lowest temperature (ESI Fig. S3†). The decrease of *α* with increasing fields, see ESI Fig. S4,† is indicative that *τ*_int_^–1^ is more sensitive to the local environment, as indeed expected. The non-monotonous trend at low temperature suggests the presence also in this powdered sample of a moderate phonon-bottleneck effect.^40^

The long spin–lattice relaxation time of VO(dpm)_2_, above 10 ms at low temperatures, suggests that also decoherence times can be comparatively long. The latter are however accessible only by pulsed EPR techniques and require narrow lines that can be achieved in diluted systems. Unfortunately, extensive efforts to prepare the titanyl-based diamagnetic analogue failed due to the instability of the mononuclear species TiO(dpm)_2_ in favour of the dimeric one [TiO(dpm)_2_]_2_,^41^ thus precluding the preparation of isomorphous crystalline solid solutions. As an alternative, two dispersions of **1** in polystyrene with mass ratio 1 : 5, **1**PS_1 : 5_, and 1 : 10, **1**PS_1 : 10_, as well as a frozen 200 mM solution of **1** in a 2 : 3 CH_2_Cl_2_ : toluene mixture (**1**sol_200 mM_), were prepared and investigated by AC susceptometry (Fig. S5†). This was aimed at getting information on the relaxation time of the magnetic susceptibility to be compared to *T*_1_ extracted from pulsed EPR data. Even if a reduced range of temperature and fields are accessible on the diluted samples for instrumental reasons, it is well evident that also diluted samples show slow relaxation of the magnetization (Fig. 2b): polymeric dispersions are characterized by a relaxation rate which is *ca.* 20 times faster than bulk sample, with minor difference between the two concentrations, suggesting that matrix effects to the relaxation dominate over those induced by dilutions. On the other hand, the low temperature relaxation rate of the frozen solution is comparable to the bulk phase but increases much faster on increasing temperatures. The almost linear temperature dependence in the log(*τ*) *vs.* log(*T*) plot of Fig. 2b allowed to analyse the data as *τ*^–1^ ∝ *T*^*n*^, with *n* = 1.49 ± 0.04 for **1**PS_1 : 5_ and *n* = 1.86 ± 0.04 for **1**sol_200 mM_. Exponents larger than one for the direct mechanism are generally attributed to spin–phonon bottleneck effects,^34^ which are expected to be more relevant for these samples that have a small contact-surface with the helium bath than the ground microcrystalline powder of the bulk sample.^40^ The field dependence of *τ* of **1**sol_200 mM_ was also investigated at *T* = 5 K (see Fig. 2c) and revealed the same wide plateau observed in the bulk phase, thus indicating that, despite some matrix effects, this interesting feature is an intrinsic property of the structure of the molecule. Unfortunately, a more direct comparison between the parameters reported in Table 1 for bulk and diluted samples would only be possible for a solid crystalline solution.

### Continuous wave and pulsed EPR spectroscopy

**Fig. 3:**
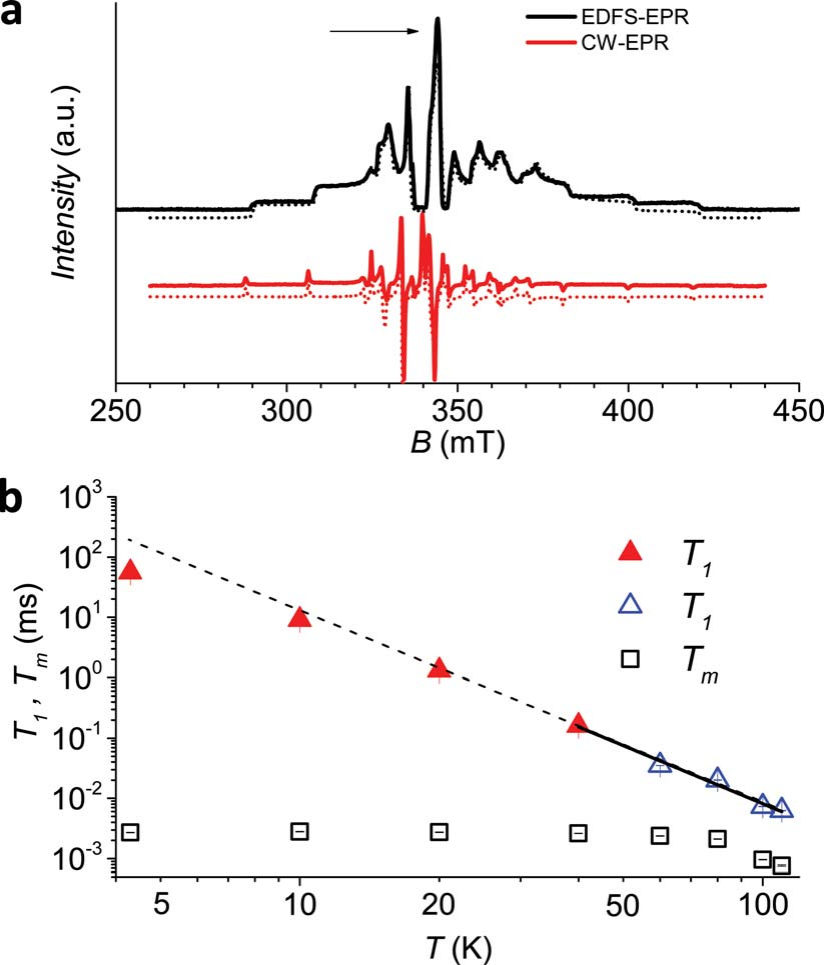
(a) Echo detected (black trace) and CW (red trace) experimental EPR spectra of **1**sol_1 mM_, measured at 5 K, and best simulations (dotted traces). The arrow marks the field position of *T*_1_ and *T*_m_ determination. (b) Temperature dependence of *T*_1_ and *T*_m_ for **1**sol_1 mM_ sample. Full symbols refer to experiments of echo saturation by fast repetition, empty ones to inversion recovery experiments (see ESI† for details). The solid line corresponds to the best-fit of the high temperature data with *T*_1_ ∝ *T*^–*n*^.

The low temperature CW-EPR X-band spectrum of a frozen 1 mM solution of **1** (**1**sol_1 mM_) is shown in Fig. 3a. Similar features are observed for the other investigated samples (**1**bulk, **1**PS_1 : 5_, **1**PS_1 : 10_, and **1**sol_200 mM_ spectra available in ESI Fig. S6†), the effect of concentration being mirrored by the narrowing of the EPR lines when going from pure sample to dispersions and frozen solutions. On the other hand, the position of the lines is not varying, indicating that the spin Hamiltonian parameters, and then the electronic structure, are maintained in different environments.

The spectra clearly show the features due to the anisotropic hyperfine coupling of the electron spin to the *I* = 7/2 nuclear spin of ^51^V: at the high and low field extreme region, peaks due to the parallel components of the hyperfine structure are observed, whereas in the centre the closely spaced perpendicular ones are evident, as schematized by the resonant fields in Fig. 1.

Spectral simulations were performed^42^ on the basis of the following spin Hamiltonian:

4 ℋ = *Î*·A·*ŝ* + *μ*_B_*ŝ*·***g***·*B*

providing as best-fit parameters: *g*_*x*_ = 1.9880(2); *g*_*y*_ = 1.9815(3); *g*_*z*_ = 1.9490(2) and *A*_*x*_ = 0.0056(1) cm^–1^ (167.9 MHz); *A*_*y*_ = 0.0063(3) cm^–1^ (190.4 MHz); *A*_*z*_ = 0.0170(2) cm^–1^ (509.6 MHz). These parameters are in the range previously reported for VO^2+^ β-diketonate-type derivatives^43^,^44^ and are consistent with the slight structural rhombicity observed by X-ray diffractometry. These spin Hamiltonian parameters have been employed to draw the Zeeman diagrams reported in Fig. 1.

An echo-detected field-swept EPR spectrum (EDFS) was recorded using the standard Hahn sequence (see ESI†) for **1**sol_200 mM_, **1**sol_1 mM,_ and **1**PS_1 : 10_ diluted samples (Fig. 3a and ESI Fig. S7†). The observation of a spin-echo is a first indication that quantum coherence is observed in these samples. Further, the same spin Hamiltonian parameters used for the simulation of the CW spectrum yielded good simulations of the EDFS spectrum, confirming that the entire VO(dpm)_2_ sample is experiencing the detected coherence.

Determination of the potential applicability of diluted samples of **1** as molecular qubit was performed by measuring the coherence time, *T*_m_, as a function of temperature and field position for **1**sol_1 mM_ to reduce spin–spin interactions. To maximize the observed echo the temperature dependence of *T*_m_ has been investigated on the so-called powder like line evidenced by an arrow in Fig. 3a.

**Fig. 4:**
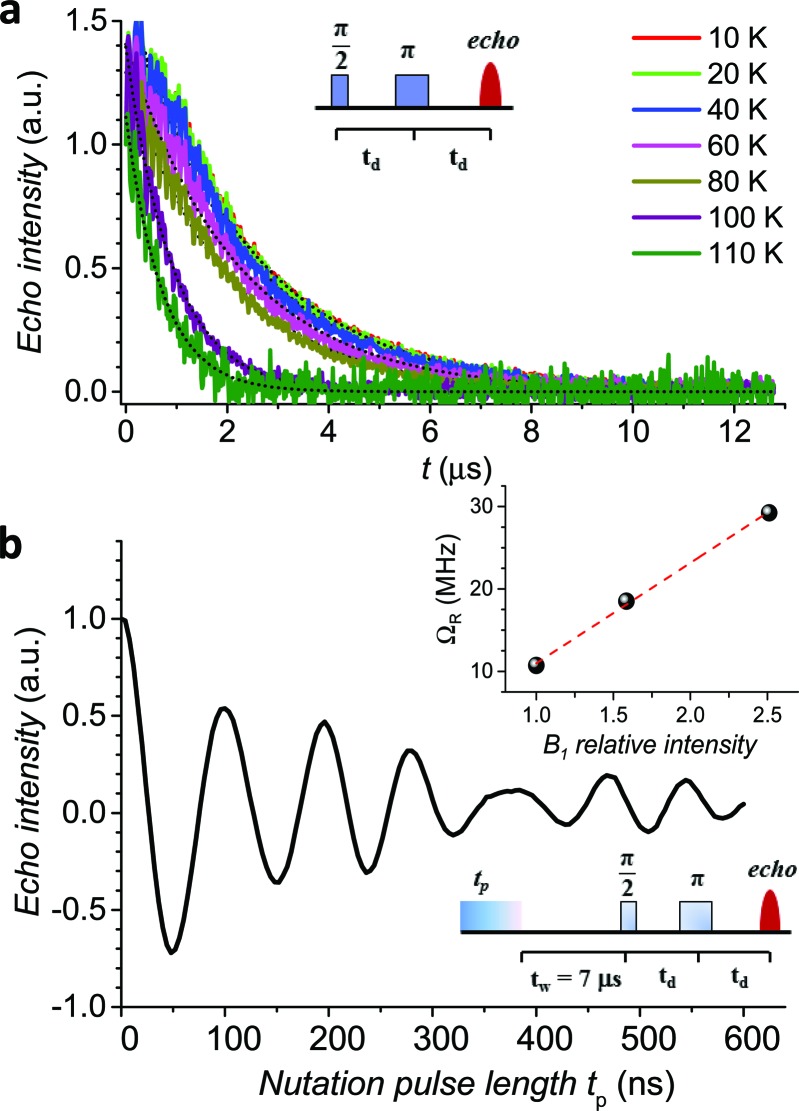
(a) Pulsed EPR Hahn echo decay traces for **1**sol_1 mM_ at different temperatures recorded at 343 mT. In the inset the employed pulse sequence. (b) Rabi oscillations for **1**sol_1 mM_ recorded at 4.3 K at 10 dB microwave attenuation. The change in oscillations observed at *t*_p_ > 400 ns is due to the interaction between the electron spin and surrounding protons.^48^ In the inset the Rabi frequency (*Ω*_R_) *vs.* oscillating field intensity superimposed to the linear best-fit.

The echo decay traces (Fig. 4a) were then fitted using a stretched-exponential equation:

5 *y* = *y*_0_ + *k*_*m*_ exp[–(2*t*/*T*_m_)^*β*_*m*_^]

It is evident that between 4 K and 80 K the resulting *T*_m_ values (Fig. 3b) are almost temperature independent (varying from 2.7 μs to 2.1 μs). Above 80 K the softening of the solvent glassy matrix, and consequently of the *tert*-butyl groups of the ligands, opens new relaxation pathways leading to the loss of echo above 110 K. The temperature dependence of *β*_*m*_ parameter essentially follows the same pattern, being slightly larger than 1 at 5 K and approaching a mono-exponential decay above 80 K (Fig. S8†). This behaviour suggests that decoherence is essentially dominated by physical motions of the magnetic nuclei.^45^

In agreement with the long coherence time observed at low temperature, Rabi-like oscillations of the echo intensity were observed for **1**sol_1 mM_ in nutation experiments performed at different microwave powers as shown in Fig. 4b, where the observed linear dependence of the Rabi oscillation on the intensity of the oscillating field is reported in the inset. This indicates the possibility of creating any arbitrary superposition of states, thus fulfilling one of the two main requirements for creating universal quantum gates.^46^

Since earlier studies revealed strong correlation between the spin–lattice relaxation time *T*_1_ and *T*_m_ for some molecular species candidate for quantum information processing (QIP),^26^,^30^ we determined the temperature dependence of *T*_1_ between 5 K and 110 K in **1**sol_1 mM_, for which the AC susceptibility technique does not have the necessary sensitivity.

Given the large range of relaxation times two different experimental procedures were applied: at low temperature (5–60 K) the echo saturation by fast repetition, suitable for long relaxation times^47^ was used, whereas at higher temperature the standard inversion recovery procedure was applied (see ESI† for details). Saturation recovery traces have been fitted using the following equation:

6 *y* = *y*_0_ + *k*_1_ exp(–*t*/*T*_1_)^*β*_1_^

with best-fit stretched parameter *β*_1_ being in the range 0.6–0.9 in the investigated temperature range (ESI Fig. S9†). The results (Fig. 3b), consistent with those previously reported by Eaton *et al.* for VO(acac)_2_ in H_2_O:glycerol solution,^43^ indicate that quite long values of *T*_1_ are observed at low temperature (50 ms at 4 K) and on heating *T*_1_ tends toward *T*_m_ (6 μs at 110 K). Interestingly, a *T*^–*n*^ dependence with *n* = 3.2 ± 0.2 is observed above 40 K, with a gradual decrease of *n* at lower temperatures, in agreement with AC susceptibility results.

To have a more quantitative comparison we also measured the temperature dependence of the spin–lattice relaxation time for **1**sol_200 mM_ by pulsed EPR: the obtained results are consistent with those obtained by AC susceptibility (Fig. 2b), confirming that the two techniques are actually probing the same process.

As a final test to establish whether this molecule maintains its long decoherence time in a solid matrix not affected by the melting of the frozen solution we measured the temperature dependence of relaxation times for **1**PS_1 : 10_. Remarkably, for this relatively concentrated sample it is possible to observe an echo and measure *T*_m_ up to 220 K (Fig. S10†). In particular while *T*_m_ is 0.36 μs at 4.3 K, *i.e.* about one order of magnitude faster than for **1**sol_1 mM_, at the highest measured temperature a *T*_m_ value of 0.1 μs could be determined.

### Deposition on Au(111) and *in situ* characterization

**Fig. 5:**
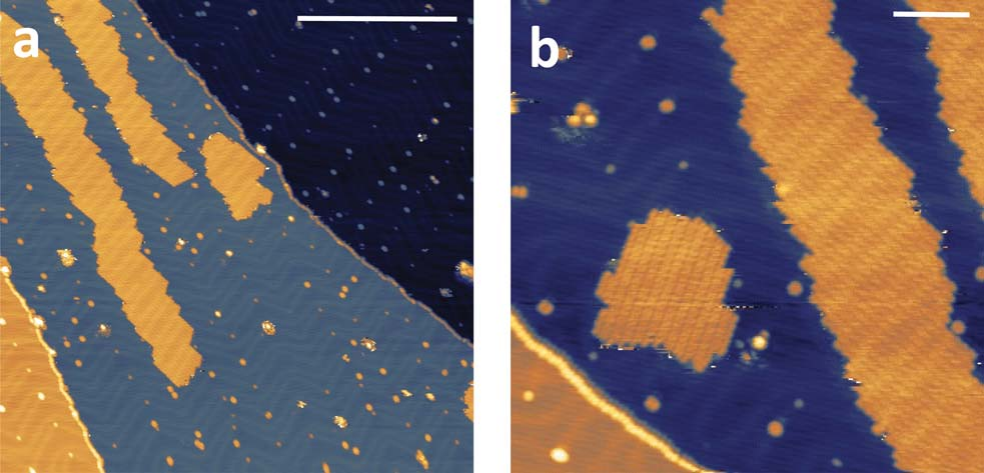
STM images of a sub-monolayer deposition of VO(dpm)_2_ measured at 30 K. Bias voltage = –2 V (empty states), tunnelling current = 5 pA. The scanned regions are 150 × 150 nm^2^ in (a) and 60 × 60 nm^2^ in (b) and the bars correspond to 50 nm and 10 nm, respectively.

The high volatility of VO(dpm)_2_ was here exploited to obtain thick films as well as sub-monolayer (ML) deposit assembled on the Au(111) surface. A complete *in situ* X-ray photoelectron spectroscopy (XPS) and low temperature scanning tunnelling microscopy (STM) characterization was carried out, while the stability of the sample toward oxidation was investigated by exposing a thick film to air. Fig. 5 reports the STM topography obtained at *T* = 30 K for sub-ML coverage. As observed for other complexes with dpm^–^ ligands,^49^,^50^ the molecules weakly interact with the substrate and form patches of variable size constituted by regularly packed molecules. The islands present regular boundaries maintaining the herringbone modulation of the gold substrate. Sub-molecular resolution was hard to achieve inside the patches, though isolated features were visible at the kinks of the herringbone structure.

The height of the molecular layer is 0.27 ± 0.02 nm (ESI Fig. S12†), in good agreement with similar deposits obtained with iron β-diketonate complex,^49^ though no reports are available on VO(dpm)_2_. By increasing the deposition time full coverage was achieved: regularly packed molecules still revealing the herringbone structure underneath were found (ESI Fig. S13†). Interestingly, as in the case of Fe(dpm)_3_,^49^ no additional molecular layer can be deposited at the employed low deposition rates (see ESI†).

**Fig. 6:**
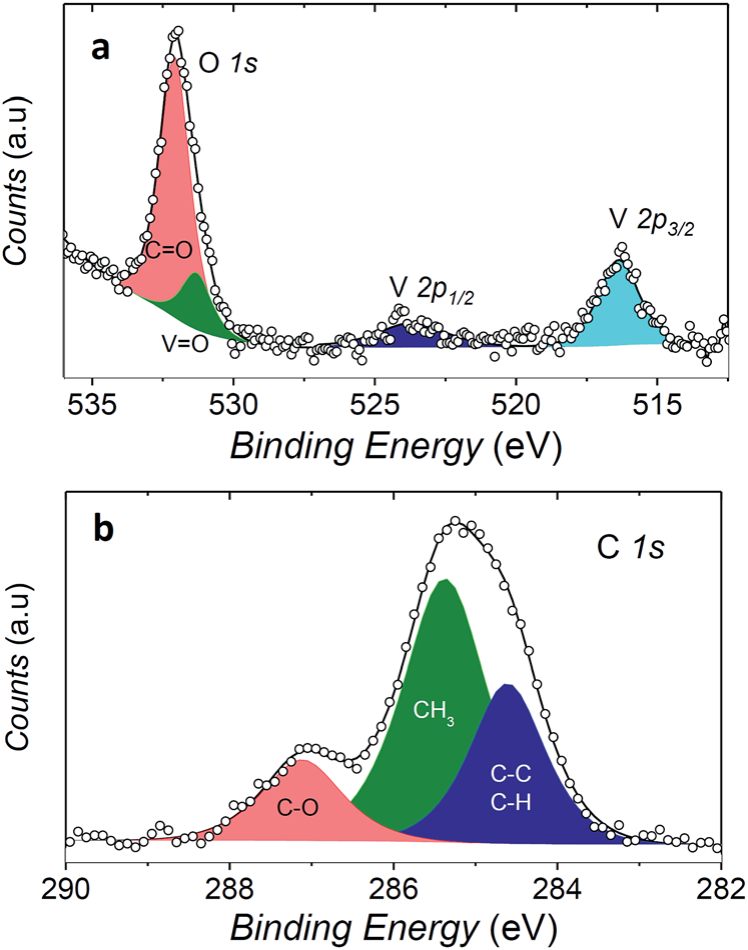
XPS spectra of a monolayer coverage of **1** on Au(111). The O 1s and V 2p regions are shown in (a) while the C 1s region in (b). Filled area and lines represent the best fit components and resulting spectra, respectively.

In order to check if the complex is intact on the surface, the full monolayer deposit was investigated by XPS, revealing the presence of the expected elements (see Fig. 6).

The observed broad O 1s peak around 532 eV was reproduced by considering two components. The smaller one at 531.3 eV was attributed to the oxygen of the vanadyl group (O_V=O_), in good agreement with what observed for vanadyl phthalocyanine, VOPc, on Ag(111),^51^,^52^ while the larger one at 532.1 eV, was associated to the oxygen atoms of the two dpm^–^ ligands (O_C=O_). The ratio of the area of the two peaks is close to 1 : 4, as expected for the stoichiometry of the molecule, thus confirming the integrity of the complex on surface. An analogous analysis allows to distinguish three components contributing to the C 1s region: the carbonylic carbon (287.2 eV), methyl carbon (285.4 eV) and the third one regrouping the remaining carbons (CH, CC, at 284.6 eV).

Even more interestingly the vanadium photoelectron peaks allowed to provide specific hints on the oxidation state of this element and thus on possible interaction with the metal surface. The V 2p_3/2_ was observed at 516.4 eV, showing a distance to the oxygen peak Δ*E*(O_V=O_ 1s – V 2p_3/2_) of 14.9 eV which well compares with that observed in VOPc monolayer and multilayers (Δ*E*(O_V=O_ 1s – V 2p_3/2_) = 14.6 eV).^51^ A semi-quantitative analysis of the composition according to the integrated peak signals gave for the three investigated elements the molar composition C = 81 ± 4%, O = 16 ± 1%, V = 2.6 ± 0.6% that well compares with the theoretical one (C = 78.6%, O = 17.9%, V = 3.6%).

These observations indicate that the VO(dpm)_2_ molecules can be deposited intact on the surface and features a weak interaction with the gold substrate as the only occupied d orbital, d_*xy*_, is expected to lie flat with limited overlap with the substrate orbitals. A similar scenario was observed for copper(ii)phthalocyanine molecules that are known to retain their unpaired electron in the d_*x*^2^–*y*^2^_ orbital.^53^ In the vanadyl derivative VOPc the metal atom is slightly above the plane of the equatorial oxygen atoms; the distance of the metal ion from the surface is therefore further increased thus reducing the interaction with the substrate. It is thus not surprising that synchrotron-based experiments on monolayers of VOPc on Ag(111)^51^ detected a substantially unchanged magnetism of the *S* = 1/2 of V^IV^ compared to thicker films. It is therefore reasonable to envisage that VO(dpm)_2_ molecules retain their paramagnetic nature when in contact with the gold substrate. This system represents therefore an appealing alternative to the use of N-donors phthalocyanine- and porphyrin-based systems for deposition on surfaces, though VO(dpm)_2_ films resulted somehow instable in air: *ex situ* prepared thick films of about 150 nm showed a partial surface oxidation as suggested by the decrease in the (Δ*E*(O_V=O_ 1s – V 2p_3/2_)) value accompanied by a progressive shift of the V 2p peak (ESI Fig. S14†).

### Comparison with other molecular spin qubits

AC-susceptometry is currently widely employed to evidence slow relaxation in SMMs characterized by easy axis anisotropy and large magnetic moments; however, Luis *et al.*^54^ have recently used this technique to characterize in detail the dynamics of a pseudo spin-doublet resulting from the large easy plane anisotropy of the *S* = 3/2 of Co^II^ in Co(acac)_2_(H_2_O)_2_.^54^ The role of hyperfine interaction and of the coupling of the nuclear spins with the phonon bath has been found to contribute to the opening of relaxation pathways otherwise forbidden in zero field for a pure *S* = 1/2 due to time reversal symmetry. VO(dpm)_2_ corresponds exactly to the hyperfine-split *S* = 1/2 model recently developed^54^ and indeed analogies in the magnetic behaviour of the two molecular systems are observed. In zero static field the hyperfine interaction with the *I* = 7/2 gives origin to two sets of states characterized by *F* = |*S* ± *I*| with multiplicity 9 and 7, respectively, as can be observed in Fig. 1. The isotropic hyperfine coupling is responsible for the gap between *F* = 4 and *F* = 3 states, which are however further split by the anisotropic components of the hyperfine tensor (see eqn (4)). The application of a weak static field has a different effect when applied along the molecular *z* direction, the one of largest hyperfine interaction corresponding to the V=O bond direction, or perpendicular to it, as also indicated in the eigenvectors composition (ESI Fig. S15†).

If similar features were already observed in the Co(acac)_2_(H_2_O)_2_*pseudo*-spin *S* = 1/2 system, some striking quantitative differences are evident. The first one is that the relaxation time remains long over a wide field range, *ca.* 30 times larger in VO(dpm)_2_ compared to the Co^II^ derivative. At 5 K, where the relaxation is still governed by the direct mechanism, the relaxation rate starts to grow above 3 T, to be compared to the drastic 0.1 T upturn observed for Co^II^ at 1.8 K.^54^ The comparison with early works on the dynamics of Cu^II^ spins^36^ confirms that the field stability of slow relaxation of VO(dpm)_2_ is unprecedented. This is extremely appealing for technological applications as it allows to exploit higher frequencies to coherently manipulate the spins, *e.g.* at W-band, corresponding to 95 GHz, with significant improvement of sensitivity. Moreover, working at W-band was also shown to increase *T*_m_ in samples of Yb^3+^ diluted in CaWO_4_, though at the same time the larger field was found to reduce *T*_1_. This serious drawback of the use of high frequencies is not expected for VO(dpm)_2_.^55^

The origin of the striking difference between the two compounds can be associated to the reduced efficiency of the direct mechanism of relaxation, which relies on the spin–phonon coupling. The latter is mediated by the spin–orbit coupling, which is significantly lower for such a light transition metal as vanadium. As a result, *T*_1_ of VO(dpm)_2_ remains long over a wide temperature range. For instance, a relaxation time of 2 ms is observed at 6 K for diluted Co(acac)_2_(H_2_O)_2_ but at temperatures as high as 40 K in concentrated VO(dpm)_2_. The anomaly arises from the small exponent of the *T*^*n*^ dependence of the Raman-like mechanism of relaxation. Such low exponents are relatively common for *S* = 1/2 states with small orbital contributions comprising light elements and have been associated to the soft character of the molecular lattices.^35^,^56^

It is also interesting to compare the measured decoherence times with those of other molecules proposed as potential molecular qubits. Among similarly investigated electron spin-qubits of vanadium, we note that the *T*_m_ values observed for a frozen solution of VO(dpm)_2_ are slightly longer – in the whole investigated temperature range – than those reported for a dispersion in protiated solvents at the same concentration of a vanadium complex with nuclear-spin free ligands:^27^ this is of particular interest, since in our case an evaporable system containing a large number of protons has been chosen without optimizing the ligand to reduce the number of nuclear spins. We have in fact confirmed that the ligand hydrogen magnetic nuclei play the dominant role in the relaxation by investigating the coherence time of VO(dpm)_2_ diluted in deuterated solvents. A substantially unchanged *T*_m_ was detected (Fig. S11†), in contrast to the improvement of at least an order of magnitude of *T*_m_^45^,^47^ expected upon solvent deuteration when nuclei flip-flops of the latter dominate the decoherence process.

The observed decoherence times for VO(dpm)_2_ are also comparable, despite the higher concentration of our samples, to those reported by Warner *et al.*^28^ for a 0.1% diluted film of CuPc (2.1 *vs.* 1 μs at 80 K, if one consider the frozen solution **1**sol_1 mM_, 0.16 *vs.* 1 μs if one consider the **1**PS_1 : 10_). We must stress that the processability and the surface stability of this β-diketonate complex are comparable to those of metal porphyrins, without the drawback of introducing ^14^N magnetic nuclei. This is not only relevant for reducing the efficiency of decoherence; as suggested by Freedman *et al.*,^27^ the well-defined hyperfine states of vanadium ions coordinated by non-magnetic nuclei can be used to investigate multiple quantum coherence. It is interesting to notice that in the Zeeman diagram of Fig. 1 pronounced level anti-crossings with gaps of the order of 0.5–1 GHz are observed at low longitudinal magnetic field. These transitions (in pale blue in Fig. 1), also known as clock transitions, are inherently robust to external perturbations because their effective *g* is practically zero and therefore are weakly affected by changes in the local field. Enhanced coherence time for these clock transitions have been recently observed in Bi doped silicon enriched in ^29^Si nuclei.^57^ Similar effects should be observable at the molecular level in VO(dpm)_2_ for which clock transitions are expected at fields where the magnetization dynamics is already rather slow.

On the other hand, when *T*_m_ of VO(dpm)_2_ is compared with relaxation times for a copper dithiolate complex with deuterated PPh_4_^+^ cation reported by van Slageren *et al.*^26^ it is found to be an order of magnitude shorter: this might be attributed both to the larger concentration of the electronic spins in our system as well as to the large number of mobile protons present on the ligand. Further, the use of Q-band frequency, as done by van Slageren *et al.*, is expected to increase *T*_m_ of VO(dpm)_2_ as well as its *T*_1_.

## Conclusions

We have shown here that a more rational search for potential qubits can significantly benefit from the combination of AC susceptometry with pulsed EPR techniques. This multitechnique approach is of particular relevance to define synthetic strategies because the optimization of *T*_1_, in terms of both temperature and field dependence, is mandatory for the realization of molecular spin qubits that can be operated at room temperature. AC susceptibility gives easily access to the field dependence of *T*_1_, in contrast to EPR, which relies on the resonance condition. Though *T*_1_ extracted with the two techniques are exactly the same only in the case of a *S* = 1/2 with no hyperfine splitting, a close relation exists also for systems with more than two levels. The simple molecule we have picked up with this approach, though not yet optimized for coherent manipulation of the spin state, presents state-of-the-art phase memory times combined with additional interesting features. The spin–lattice relaxation remains slow even in strong fields, allowing the use of higher frequencies for coherent spin manipulation without losses in performances.

A particularly low efficient spin–phonon coupling appears to be at the basis of this behaviour and the potentially positive role played by the strong V=O bond needs to be further investigated by extending the approach developed here to other and more promising systems.^27^,^29^ *Ab initio* modellization of the spin relaxation could also help to identify which structural features can favour long *T*_1_, and consequently long *T*_m_, at high temperature.

Even if the crucial aspect of qubits entanglement has not been addressed in this work it can be easily achieved through connection of β-diketonate pockets in more complex architectures.^58^,^59^ This approach has already been successfully employed to couple different spin centers^60^ and to address them individually in resonance experiments in particular in lanthanide polynuclear complexes thanks to their significantly different ***g***-factors.^61^

Of great relevance is the possibility to obtain monolayers of ordered arrays of intact VO(dpm)_2_ molecules, retaining their paramagnetic nature thanks to the reduced interaction of the orbital carrying the unpaired electron with the substrate. Metallic nanostructures can be easily decorated with a monolayer of ordered VO(dpm)_2_ molecules, allowing to investigate the response of an ensemble of identical molecular qubits, whose size can be easily controlled by lithographic exposure of the metallic substrate. Thin films of VO(dpm)_2_ could be evaporated directly on a μ-SQUID to detect by AC susceptometry the effects of surface confinement on the dynamics of the magnetization, as already done on SMMs.^62^ Our low temperature STM investigation suggests that VO(dpm)_2_ could be also a good candidate to investigate quantum coherence at the single molecule level thanks to the recently developed approach based on spin-polarized scanning tunnelling microscopy, employed at very low temperature on single Fe atoms deposited on a MgO surface.^63^

Combining the optimization of *T*_2_ in nuclear spin free environments with the possibility to control the spin–lattice relaxation through a rational synthetic design is foreseen to boost the interest for molecular spin systems as potential qubits.

## Supplementary Material

Supplementary information
