# Supplementary material for: Quantum coherence in a processable vanadyl complex: new tools for the search of molecular spin qubits
Source: Chem Sci. 2015 Dec 11;7(3):2074–83. doi: 10.1039/c5sc04295j (PMC5968563; doi:10.1039/c5sc04295j)
Supplement: Supplementary file 1 [file SC-007-C5SC04295J-s001.pdf]

Electronic Supplementary Material (ESI) for Chemical Science.
This journal is © The Royal Society of Chemistry 2015

Electronic Supplementary Information

Quantum Coherence in a processable vanadyl complex: new tools for the search of molecular spin qubits

Lorenzo Tesi[1], *Eva Lucaccini*[1], *Irene Cimatti*[1], *Mauro Perfetti*[1], *Matteo Mannini*[1], *Matteo Atzori*[1], *Elena Morra*[2], *Mario Chiesa*[2], *Andrea Caneschi*[1], *Lorenzo Sorace*[1*], *Roberta Sessoli*[1*]

1) Department of Chemistry "Ugo Schiff", University of Florence & INSTM RU of Florence, via della Lastruccia 3-13, 50019 Sesto Fiorentino, Italy

2) Department of Chemistry, University of Turin & NIS Centre, via P. Giuria 7, 10125, Torino, Italy

* Correspondence: lorenzo.sorace@unifi.it, roberta.sessoli@unifi.it

Synthesis and samples preparation. The whole synthesis was performed in inert atmosphere following a reported procedure.[1] Vanadium sulphate tetrahydrate (0.5 g, 2.13 mmol) was dissolved in 4 ml of H_2O milliQ and 8 ml of EtOH with magnetic stirring. Hdpm (1 g, 5.45 mmol) was added and the solution quickly became dark green. Finally, 3 ml of sodium carbonate 0.1 M was mixed with the solution and a solid appeared immediately. The latter was filtered out and washed with water. The powder was purified by sublimation thanks to which we obtained long green crystals. Anal. Calcd. (found) for [$C_{22}H_{38}O_5V$]: C, 60.96 (61.07); H 8.84 (9.00). Crystals were checked by X-ray diffraction and resulted to correspond to the structure available in the Cambridge Structural Database record CCDC 230339.[1]

Diluted samples were also prepared to evaluate the effect of intermolecular interactions on magnetization dynamics. Glassy dispersions were obtained by dissolving $VO(dpm)_2$ in a 2:3 toluene:CH_2Cl_2 solution to obtained a glass at low temperature or by dispersing the complex in polystyrene films.

AC susceptometry. A Quantum Design PPMS equipped with AC susceptibility probe (working in the range 10 Hz- 10 kHz) and a Quantum Design MPMS SQUID magnetometer (0.1 Hz - 1 kHz), have been used to measure the magnetic susceptibility over an extended frequency. The higher sensitivity of the latter set-up allowed to characterize the diluted samples. A variable static field was applied parallel to the oscillating field.

Through the Debye model (equation 1) we extrapolated the relaxation time, τ, and the width of the distribution of the relaxation time, α.

$$\chi''(\omega) = (\chi_T - \chi_S)\frac{(\omega\tau)^{1-\alpha}\cos\left(\frac{\pi\alpha}{2}\right)}{1 + 2(\omega\tau)^{1-\alpha}\sin\left(\frac{\pi\alpha}{2}\right) + (\omega\tau)^{2-2\alpha}} \qquad \text{(eq.1)}$$

where $\chi''(\omega)$ is the imaginary susceptibility, ω the angular frequency, χ_T the isothermal susceptibility and χ_S the adiabatic susceptibility.

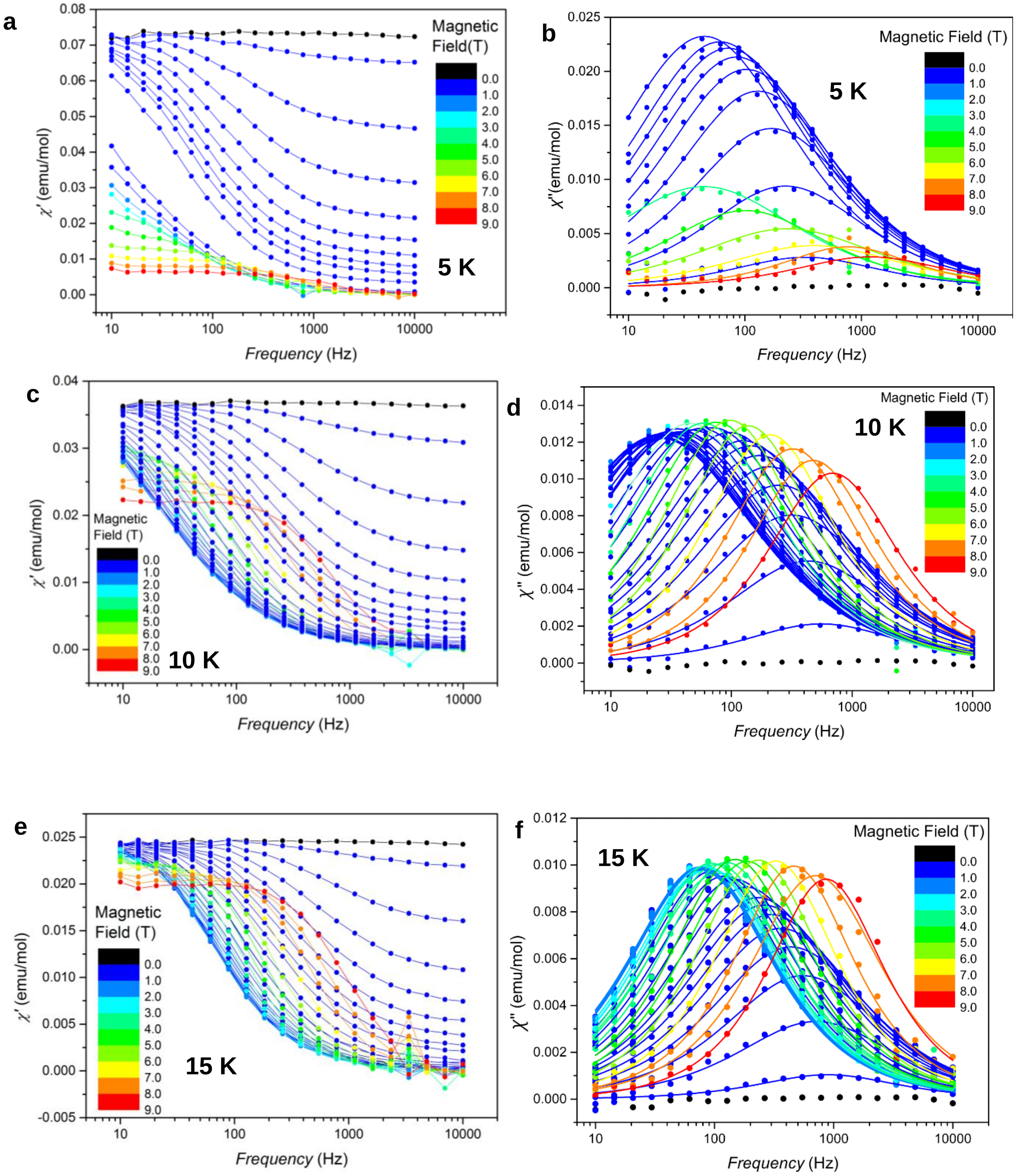

Figure S1. *Frequency dependence of the real (χ') and imaginary (χ'') susceptibility of VO(dpm)$_2$ bulk with static magnetic fields varying between 0 and 8.8 T. (a) and (b): at 5 K; (c) and (d): at 10 K; (e) and (f): at 15 K.*

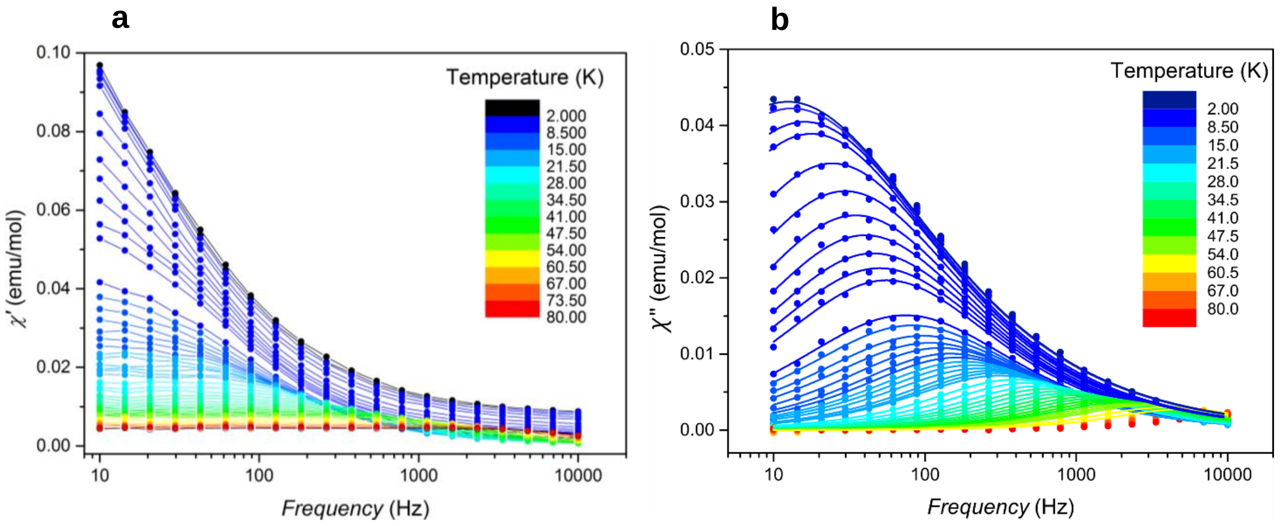

Figure S2. *Frequency dependence of the real susceptibility χ' (a) and of imaginary susceptibility χ'' (b) of VO(dpm)$_2$ bulk as a function of temperature between 1.8 and 80 K in a static applied field of 0.2 T.*

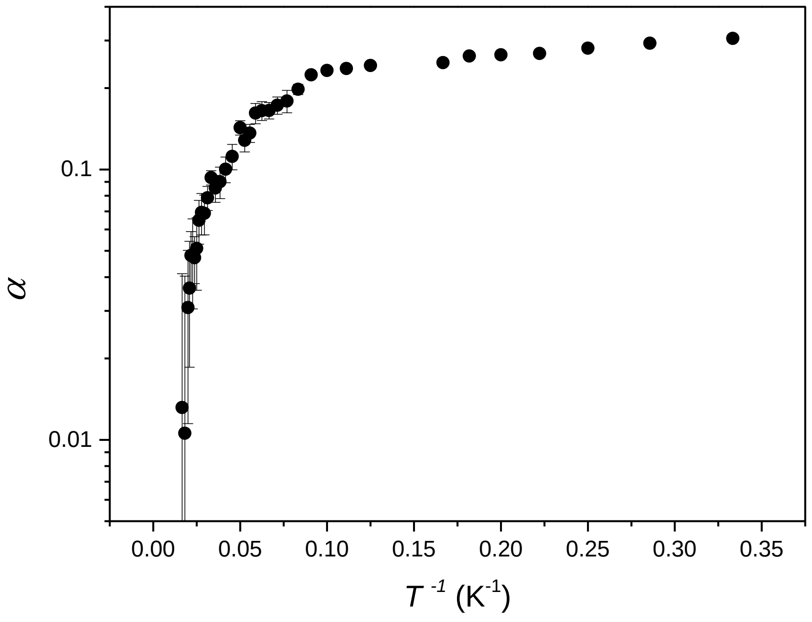

Figure S3. *Width of the distribution of the relaxation time for VO(dpm)$_2$ bulk, extrapolated by the Debye model through the α parameter, as a function of the reciprocal temperature in B_{dc}=0.2 T.*

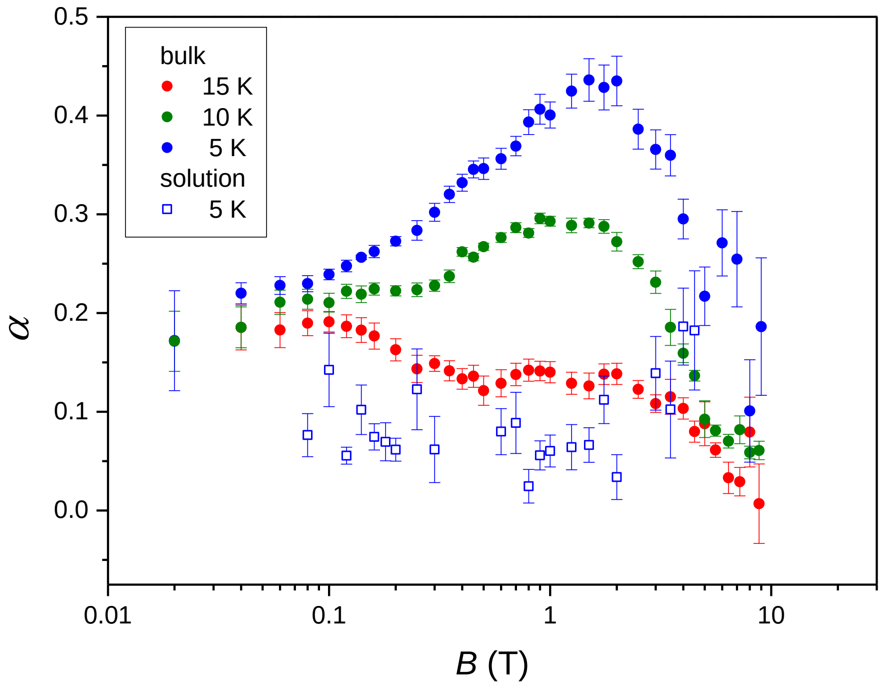

Figure S4. *Width of the distribution of the relaxation time, extrapolated by the Debye model through the α parameter, as a function of the magnetic field, both for the bulk at 5, 10, 15 K, and for the 200 mM solution of VO(dpm)$_2$ at 5 K.*

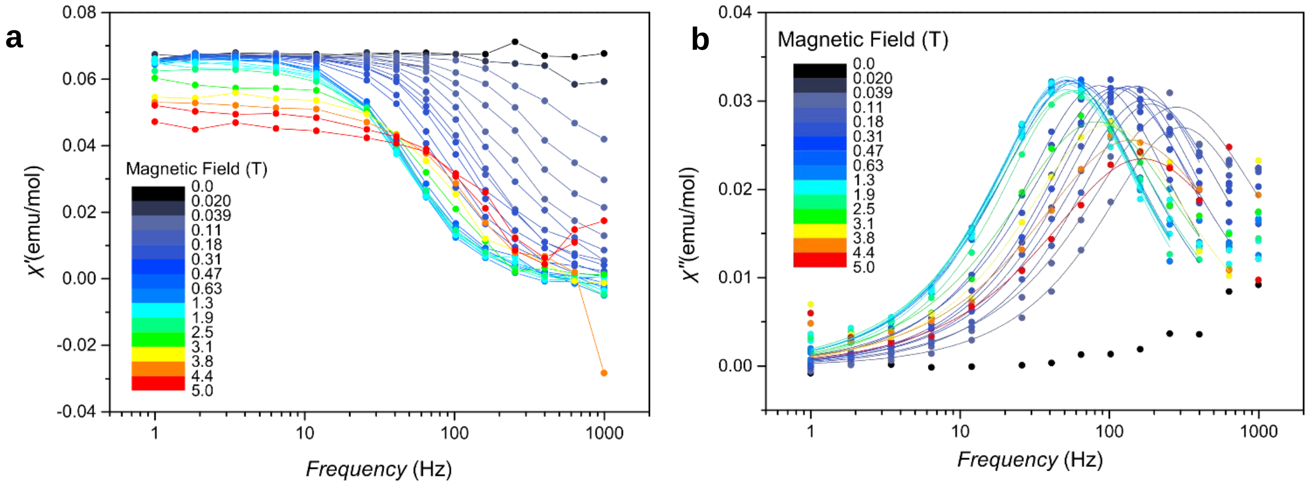

Figure S5. *Frequency dependence of the real susceptibility χ' (a) and of imaginary susceptibility χ'' (b) of a 200 mM VO(dpm)$_2$ solution in CH_2Cl_2-toluene 1:2 as a function of the magnetic field between 0 and 5 T at 5 K.*

EPR Spectroscopy. CW X-Band EPR spectra of all samples were recorded on a Bruker Elexsys E500 spectrometer equipped with a SHQ cavity (ν = 9.47 GHz, Florence). Low temperature measurements were obtained using an Oxford Instruments ESR900 continuous flow helium cryostat. Pulsed EPR measurements (**1**$PS_{1:10}$, **1**$sol_{200mM,}$ **1**$sol_{1mM,}$ **1**sol_{1mM}^{D}) were performed with a Bruker Elexsys E580 at X-band (Turin, ν = 9.75 GHz) equipped with a flexline dielectric ring ENDOR resonator (Bruker EN 4118X-MD4). Temperatures between 4 and 200 K were obtained with an Oxford Instruments CF935 continuous flow helium cryostat. Typical pulse lengths were 16 ns (*π/2*) and 32 ns (*π*). For Echo detected field swept EPR spectra, the Hahn Echo pulse sequence (π/2—t_d—π— t_d—echo), with fixed interpulse delay time t_d=200 ns, was applied while sweeping the magnetic field.

Phase memory times measurements were obtained by measuring the primary echo decay with varying interpulse delay starting from t_d=98 ns at a fixed magnetic field. Spin–lattice-relaxation times were measured using the standard inversion recovery sequence (π—t_w—π/2— t_d—π— t_d—echo) and by observing the variation of the amplitude of the primary echo as a function of the repetition rate (echo saturation by fast repetition). This second method was find to be more convenient at low temperature (T<40 K) due to the very long T_1 of the sample. Nutation measurements were performed with a nutation pulse (t_p) of variable length followed by a Hahn echo sequence (t_p-t_w—π/2— t_d—π— t_d—echo) with t_w =7 μs, t_d=200 ns and different pulse powers. The pulse length varied depending on the attenuation level of B_1. For 2 dB attenuation, the second and third pulses used were 10 and 20 ns in length. For 6 dB attenuation, 16 and 32 ns pulse lengths were used, and for 10 dB attenuation the second and third pulses were 26 and 52 ns in length respectively.

CW and Echo-detected spectra were simulated with Easy-Spin.[2] Errors of fit parameters (*g* and hyperfine values) were estimated by eye. T_m relaxation data were normalized to the first measurement point and fitted with Origin; indicated deviations correspond to the standard errors. Phase memory times (T_m) were extracted from fitting (stretched) exponentials, equation (4), to the Hahn echo decay curves. Experimental data of longitudinal relaxation times were fitted with a stretched exponential decay, equation (5).

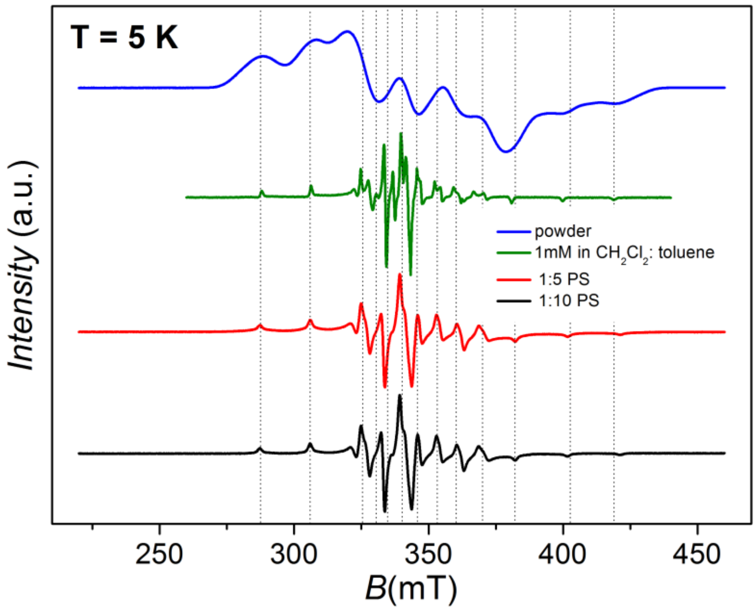

Figure S6. *CW-EPR X-band spectra at 5 K for the bulk sample, the PS dispersion (1:5 and 1:10) and the frozen 1mM solution of VO(dpm)$_2$. The dotted lines evidence the coincidence of the resonant fields in the different samples.*

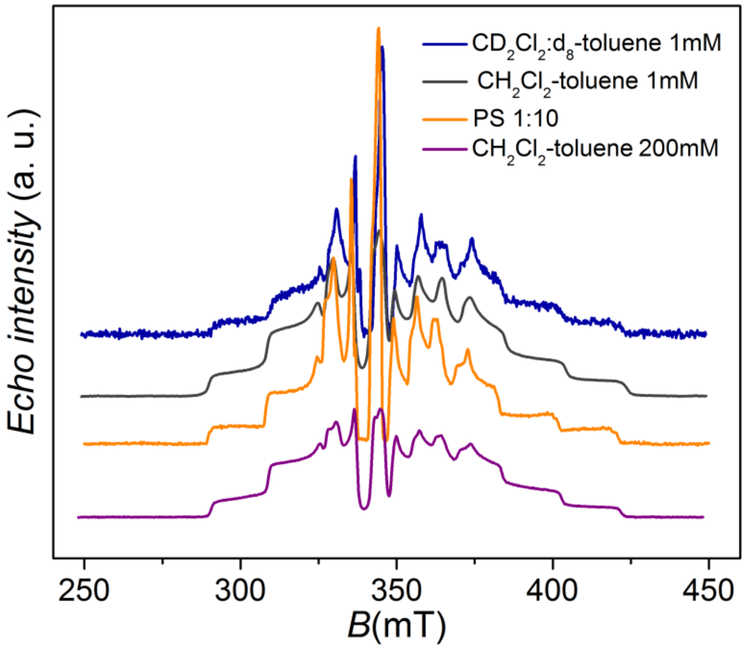

Figure S7. *Echo detected field swept EPR spectra of VO(dpm)$_2$ dispersed into PS (1:10), in a 1 mM deuterated solution, in a 1 mM protic solution and in a 200 mM protic solution. All the spectra were recorded at 5 K, except for the concentrated solution one, that was measured at 10 K due to receiver saturation at 5 K.*

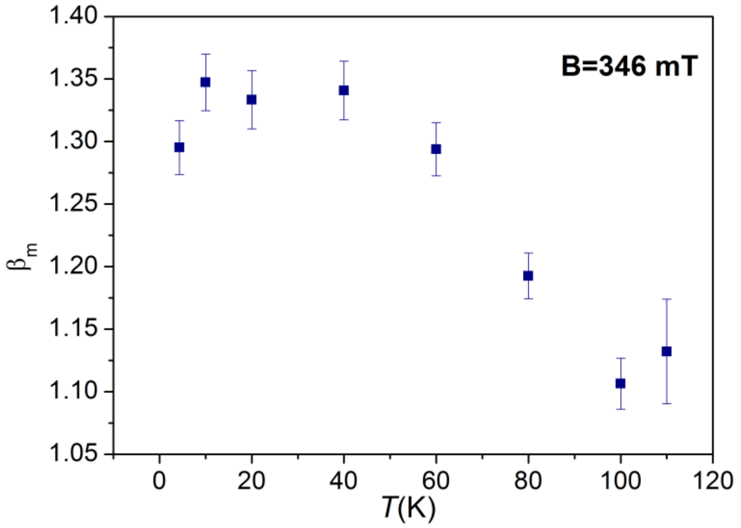

Figure S8. *Temperature dependence of the best fit values of the stretching parameter* β_m *for* $VO(dpm)_2$ *in a 1 mM* CH_2Cl_2*-toluene frozen solution.*

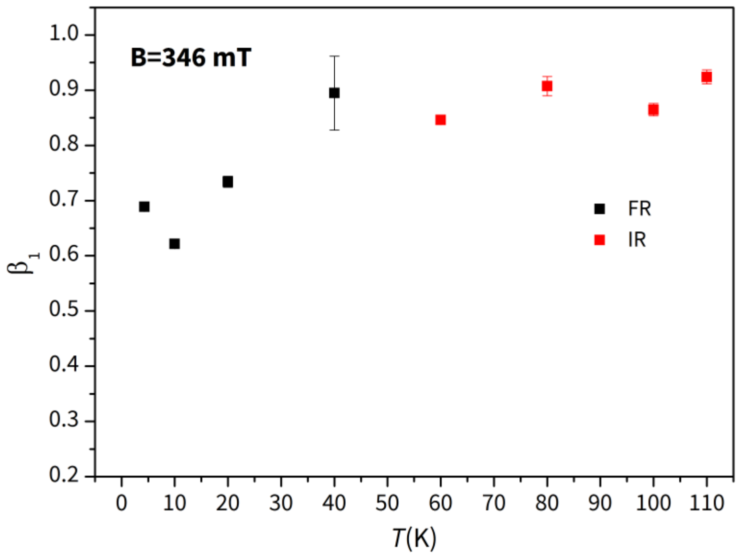

Figure S9. *Best fit values of the stretching parameter* β_1*, as a function of temperature for* $VO(dpm)_2$ *in a 1mM* CH_2Cl_2*-toluene frozen solution. The spin-lattice relaxation time was measured with echo saturation by fast repetition for low temperatures (black squares) and with inversion recovery mode for higher temperatures (red squares).*

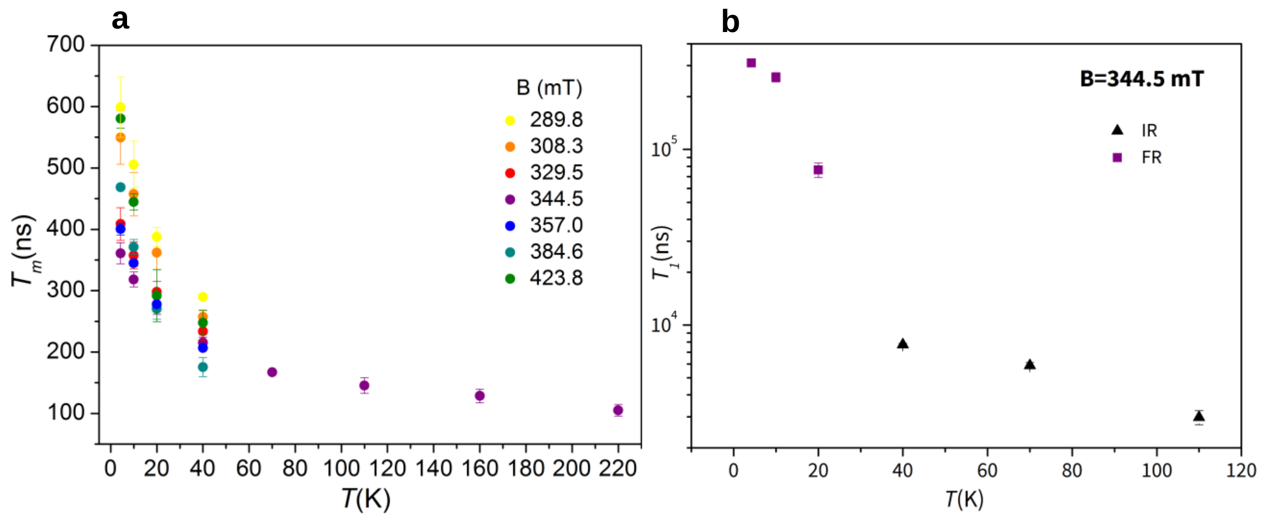

Figure S10. *a) Temperature dependence of T_m values for VO(dpm)$_2$ dispersed in PS (1:10), evaluated at resonant fields corresponding to parallel and perpendicular transitions between different hyperfine sublevels (B=289.8 mT: parallel, m_I=-7/2; B= 308.8 mT parallel m_I=-5/2; B=329.5 mT perpendicular m_I=-5/2; B=357.0 mT perpendicular m_I= 5/2; B=384.6 mT parallel m_I=3/2; B=423.8 mT parallel m_I=7/2). b) Comparison of T_1 values of the central line measured with echo saturation by fast repetition (purple squares) and inversion recovery mode (black triangles).*

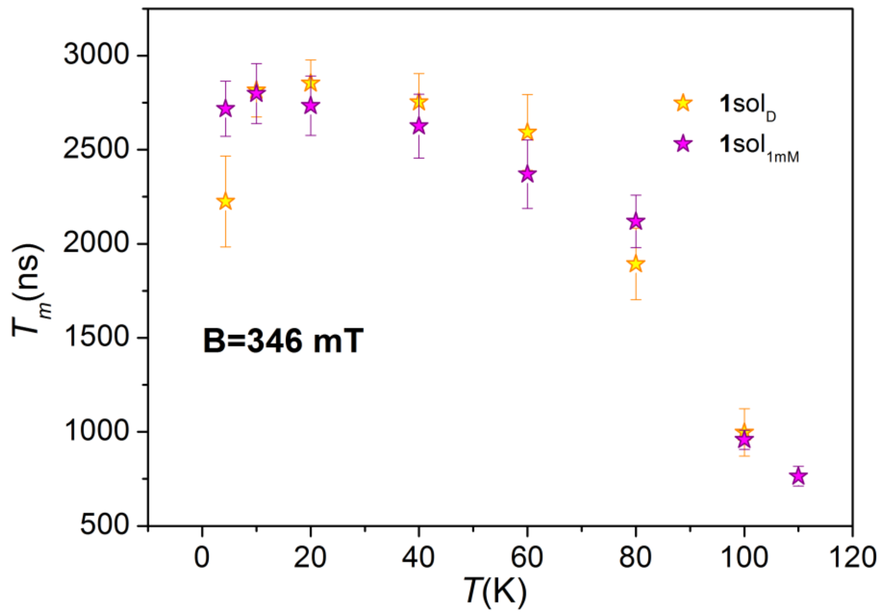

Figure S11. *Comparison of the phase memory time T_m measured at 346 mT for protic and deuterated 1mM solution of VO(dpm)$_2$.*

UHV deposition and characterization. The ML and sub-ML deposition and characterization of $VO(dpm)_2$ films were performed *in situ*. The substrate employed was an Au(111) single crystal. The surface was cleaned by repeated Ar^+ sputtering (2 μA, 1 keV) and annealing (720 K) cycles. Considering that $VO(dpm)_2$ like other β-diketonates shows high volatility,[3] the sublimation was performed in a dedicated preparation chamber with a base pressure of 1×10^{-7} mbar; this chamber is directly connected to the XPS and STM chambers. Low coverages were obtained by keeping the molecular powders, hosted in a quartz crucible, at room temperature. The comparison of the STM and XPS characterization of an *in situ* monolayer deposition performed by heating the powders (at 373 K, where a rate is observed by QCM) or leaving them at room temperature, proves that there is no difference between the two. During the sublimation, the substrate was kept at room temperature. A K-type thermocouple, buried into the molecular powder, allowed for temperature control.

The thick film was prepared in a home-made evaporation chamber, and transferred to the XPS chamber using a glove bag filled with nitrogen. The sublimation was performed on top of a polycrystalline Au film evaporated on Mica. Preliminarily to the sublimation a hydrogen flame-annealing procedure was adopted in order to clean the *ex situ* prepared substrate. The deposition was performed using the same evaporator as for the monolayer coverage but the powders were heated at 373 K.

The STM images were obtained by an UHV scanning tunneling microscope (Omicron VT-STM) operating at 30 K in the constant current mode with electrochemically-etched W tips. The applied tip bias voltage and the tunneling current of each image are given in the figure caption.

The height estimation of the $VO(dpm)_2$ molecules was carried out by plotting the height distribution of selected regions (see highlighted areas Figure S12). The measured heights of each region were then fit with two Gaussian functions, one for molecular domains and a second for the background. The height of the molecular domain is estimated as the difference between the peak positions of the two distributions. We then computed the mean height value averaging over the five considered areas. The error on the mean value, *s*, is then given by

$$s = \sqrt{\frac{\sum_{k=1}^{N} \sigma_k^2}{N-1}}$$

where σ_k is the standard deviation of height computed for the k-th area and N is the total number of the considered values.

XPS measurements were carried out in an UHV chamber with a base pressure in the low 10^{-10} mbar range. The chamber is equipped with a SPECS Phoibos 150 electron analyzer, a standard Al source

and a monochromatic Al X-ray source. The X-ray sources were assembled at 54.44° with respect to the analyzer. For the characterization of the monolayer deposition, we used the monochromatic Al source operating at a power of 100 W (13 kV and 7.7 mA). The characterization of the thick film was performed with a standard Al source with a power of 260 W (13 kV and 20 mA). The pass energy was set to 40 eV for all the experiments.

The monolayer was subjected to differential charging and the Au $4p_{3/2}$ peak present the same shift as the main ones of the molecule (O *1s*, V *2p*, C *1s*). Performing the calibration using the Au $4p_{3/2}$, the position of the main peak of C *1s* was 285.4 eV. In order to use the same calibration for the thin and the thick film, being the Au *4p 3/2* not visible, the methyl C *1s* at 285.4 eV signal has been used as a reference to correct the charging effect.

XPS data analysis have been performed by removing the inelastic background by means of the Shirley method[4] and then deconvoluting the experimental spectra using mixed Gaussian and Lorentzian line shapes for each component in the spectra. In the case of V *2p* component the adopted method resulted in line with previous literature reports.[5] The background for the O *1s* peak was obtained by subtracting also the contribution of the Au *4p 3/2* peak at 547 eV.

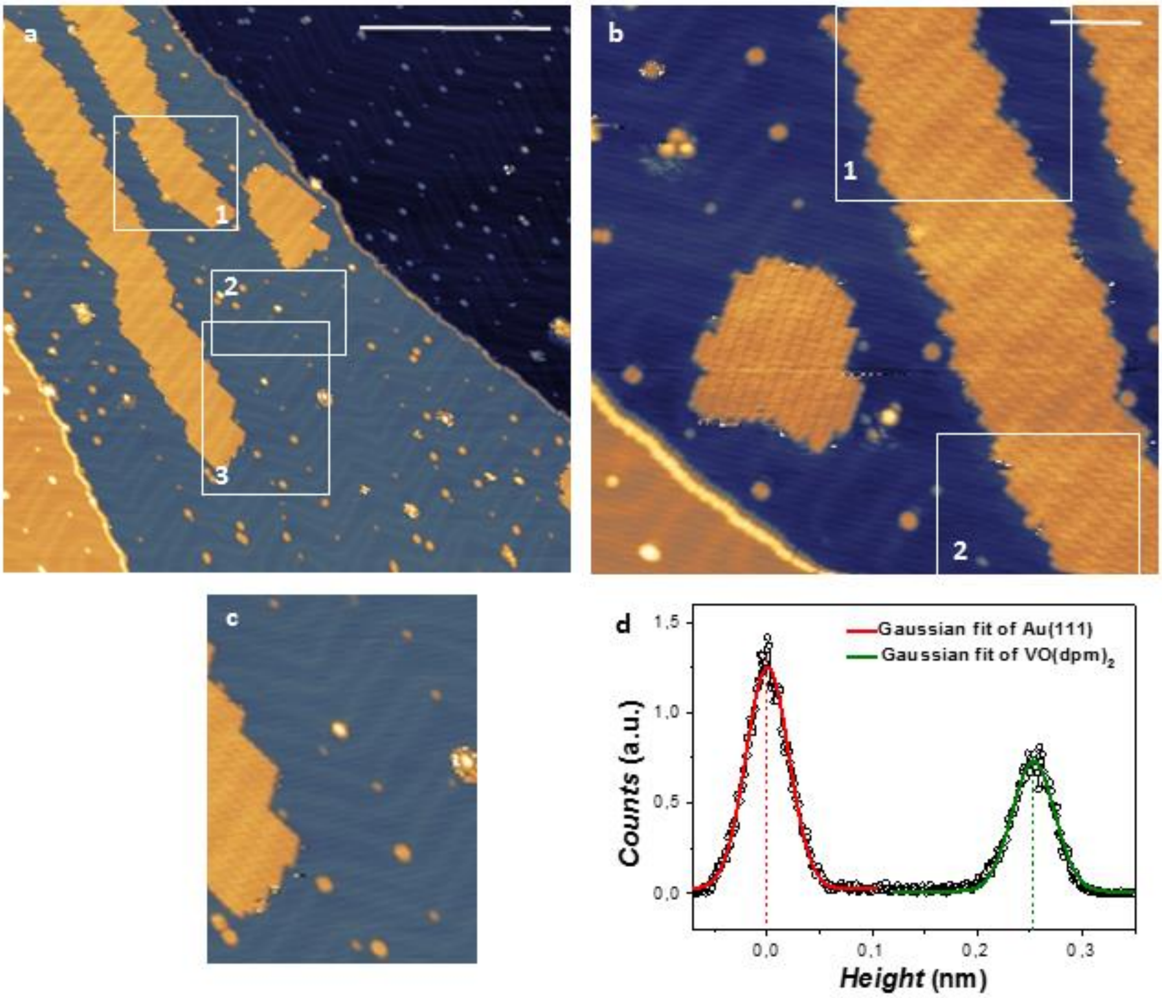

Figure S12. *a) and b) are STM images of a sub-monolayer deposition of VO(dpm)$_2$ measured at 30K, already reported in Fig. 5. The zones into the frames are the ones used for the evaluation of the height distribution. c) is the enlarged view of zone 3 in a), and d) is its height distribution.*

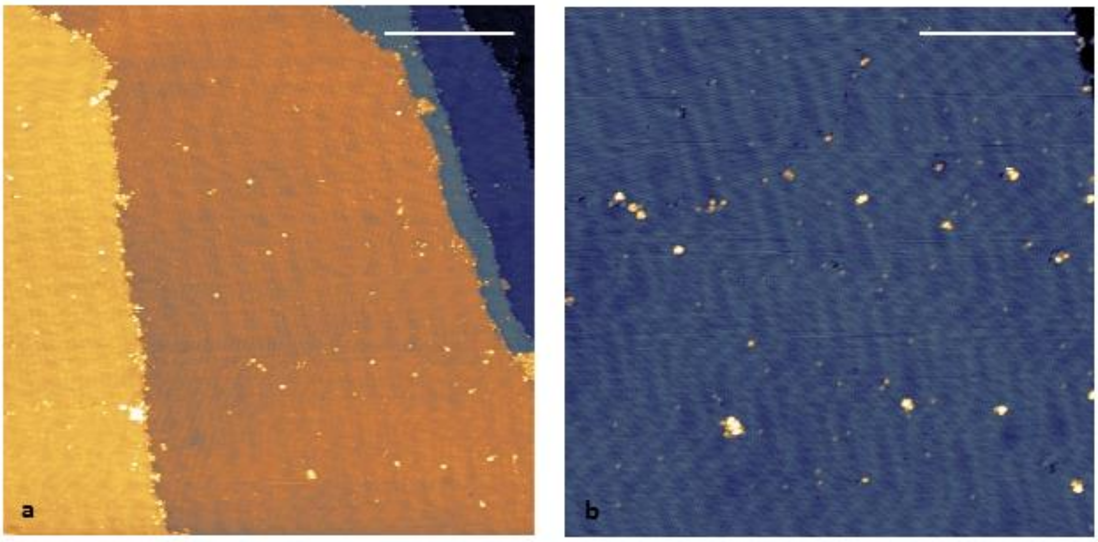

Figure S13. *STM image of a monolayer deposition of VO(dpm)$_2$ measured at 30 K. Bias = -2 V, current = 5 pA. a) 200x200 nm region. b) 100x100nm region, zoom of the precedent. The scale bar are 50 nm (a) and 30 nm (b).*

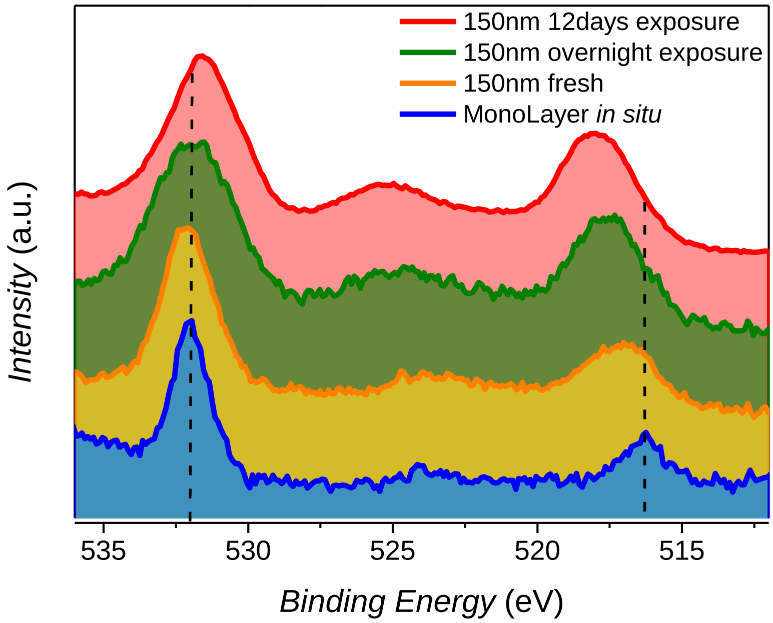

Figure S14. *Comparison of O 1s and V 2p XPS spectra of the monolayer and thick film. The thick film of 150 nm was prepared ex situ and transferred into the XPS chamber using a glove bag filled with nitrogen; the monolayer was prepared and kept in situ. The thick film was exposed to air for a variable time reported in the legend.*

Compositions of the eigenstates of the *S*=1/2, *I*=7/2 coupled system.

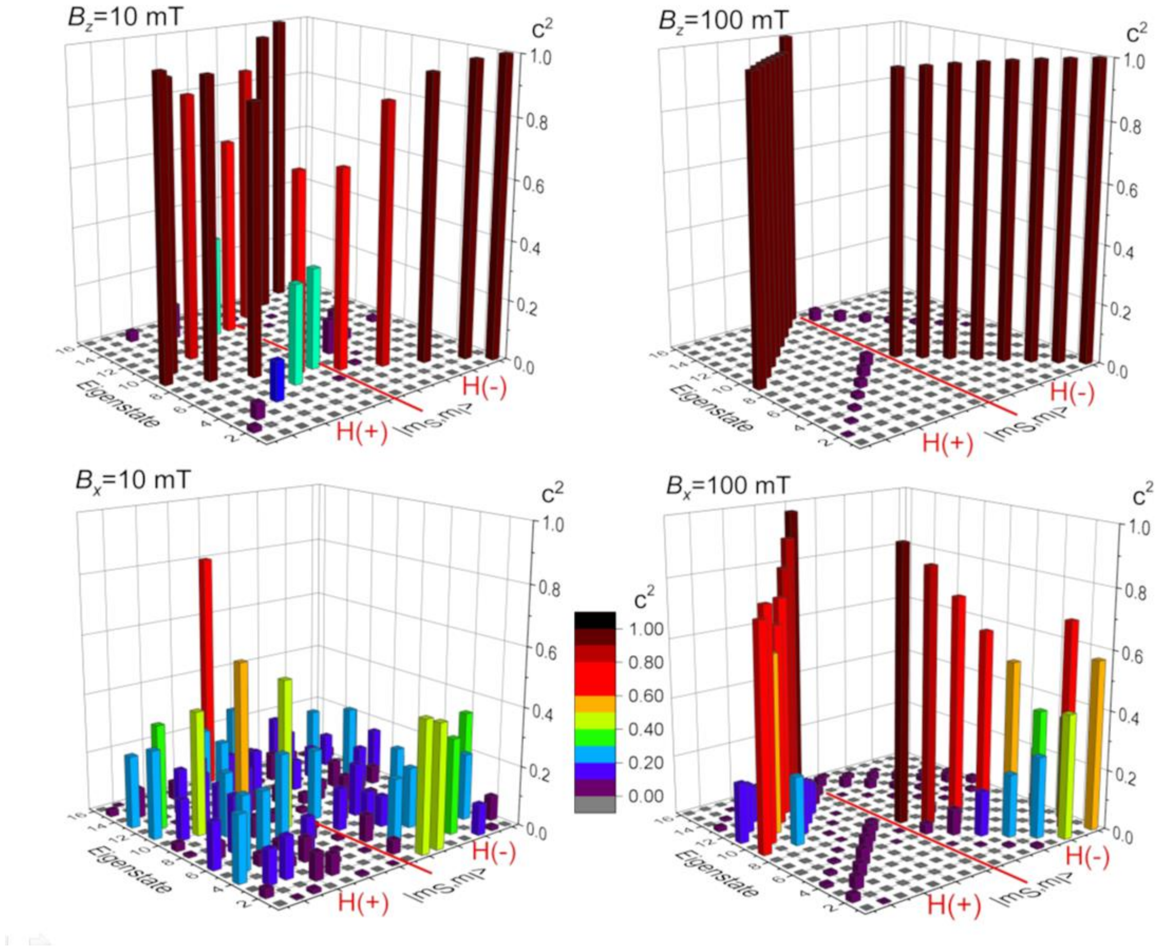

Figure S15. *Eigenstates composition at two different magnetic fields (10 and 100 mT) applied parallel and perpendicular to the largest hyperfine coupling component calculated with the spin Hamiltonian of eq. 3 of main text (see text for parameters). The basis has been selected as the projection along the field direction. The red lines separate basis-states with projection of the spin parallel (H+) and antiparallel (H-) to the external field.*

Bibliography

S1 Ahmed, M. A. K.; Fjellvåg, H.; Kjekshus, A.; Klewe, B. *Z. Anorg. Allg. Chem.* **2004**, 630, (13-14), 2311-2318.

S2 Stoll, S.; Schweiger, A. *J. Magn. Reson.* **2006**, 178, (1), 42-55.

S3 Evans, S.; Hamnett, A.; Orchard, A. F.; Lloyd, D. R. *Far. Disc. Chem. Soc.* **1972**, 54, 227-250.

S4 Shirley, D. *Phys. Rev. B* **1972**, 5, 4709-4714.

S5 Biesinger, M. C.; Lau, L. W. M.; Gerson, A. R.; Smart, R. S. C. *Appl. Surf. Sci.* **2010**, 257, 887-898.
